# The Development of an Unmet Needs Appraisal Scale for Long-Term Care Service Users With Disability Certificates

**DOI:** 10.1093/geroni/igaf122.3038

**Published:** 2025-12-31

**Authors:** Peng-Li Wang, Shih-Cyuan Wu, Hsiao-Wei Yu, Shwu-Chong Wu, Shwn-Jen Lee, Shu-Chen Lu, Shiau-Fang Chao, Ya-Mei Chen

**Affiliations:** National Taiwan University, Taipei, Taipei, Taiwan (Republic of China); National Taiwan University, Taipei, Taipei, Taiwan (Republic of China); National Taiwan University, Taipei, Taipei, Taiwan (Republic of China); National Taiwan University, Taipei, Taipei, Taiwan (Republic of China); National Yang Ming Chiao Tung University, Taipei, Taipei, Taiwan (Republic of China); Taiwan Occupational Therapy Association, Taipei, Taipei, Taiwan (Republic of China); National Taiwan University, Taipei, Taipei, Taiwan (Republic of China); National Taiwan University, National Taiwan University, Taipei, Taiwan (Republic of China)

## Abstract

In 2017, Taiwan launched Long-Term Care (LTC) Plan 2.0, expanding services to include those under 49 with disability certificates. The LTC unmet needs of this group can be different from those general LTC users. By 2021, 182,555 people with disability certificates (51% of LTC users) had received LTC services. The Unmet Needs Appraisal Scale (UNAS) for general LTC users, developed by Professor YM Chen’s team, is a tool with one domain and 11 items. The purpose of this study was to develop UANS for People with Disability Certificate (UNAS-Disability People) . After expert panel, three items were added to the tool with 14-item version of the UNAS- Disability People. The revised tool was pilot tested in collaboration with city governments and NGOs associated with various types of disabilities. We examined the tool’s properties, including reliability and validity, and included Short-Form 8 as a measure of concurrent validity. From northern and central Taiwan, 620 questionnaires were collected. The UNAS-Disability People initially had 14 items across two domains: unmet needs for mental and physical health (Cronbach’s α = 0.91). Ultimately, 13 items and two domains were retained, showing good reliability and validity [χ²=384.582, df = 64, p<.0001; CFI=0.906; RMSEA=0.085; SRMR=0.051]. The two domains were moderately correlated (r = 0.69, p<.0001). Both domains showed moderate negative correlations with Physical Component Summary and Mental Component Summary (r = -0.450, p < .0001; r = -0.414, p < .0001), confirming good concurrent validity. The UNAS-Disability People can provide better LTC unmet needs assessment for people with disability certificates with two domains of unmet needs.

